# Progresses in Questing for the Truth of Opioid‐Related Constipation in Cancer Patients

**DOI:** 10.1111/jcmm.70553

**Published:** 2025-04-25

**Authors:** Mengxue Liu, Yedong Sheng, Yingrong He, Shixiang Wu, Chunhui Jin, Lijuan Shen

**Affiliations:** ^1^ Wuxi Hospital Affiliated to Nanjing University of Chinese Medicine Wuxi Jiangsu China; ^2^ Nanjing University of Chinese Medicine Nanjing Jiangsu China

**Keywords:** cancer, constipation, enteric nervous system, intestinal cells of Cajal, intestinal flora, opioid

## Abstract

Opioids are extensively utilised to manage pain in cancer patients, but may cause constipation which significantly impacts their prognosis and quality of life. Opioid‐induced constipation (OIC) lacks effective drugs and management strategies. Opioids act on the enteric nervous system, intestinal barrier, intestinal immunity and intestinal microbiota, implying that OIC is a multifactorial process. This paper aims to examine the effects of opioids on the intestine, specifically the enteric nervous system, intestinal barrier and interstitial cells of Cajal (ICCs), and elucidate the primary mechanisms underlying OIC development and deterioration. This review suggests that enteric neurons, intestinal immunity and intestinal flora could serve as potential therapeutic targets for OIC.

## 1 Introduction

Both the incidence and mortality rates of malignant tumours, a leading cause of death, are on the rise in China [1]. Pain appears in 69% of cancer patients [2], and the use of opioids causes OIC in up to 60%–90% of these patients [3]. The pathogenesis of OIC is complex, making it challenging to develop effective therapeutic interventions. Therefore, this review aims to delve into the pathogenesis of OIC, identify potential therapeutic targets and propose research directions.

## 2 Definition and Diagnostic Criteria for OIC

OIC has been regarded as a symptom of opioid intestinal dysfunction (OIBD), rather than a disease [4]. However, increasing evidence supports the categorisation of OIC as a distinct disease that necessitates interdisciplinary management involving gastroenterologists, oncologists, geriatricians and pain specialists [5, 6]. Currently, the Rome criteria are widely accepted to diagnose OIC: new or worsened constipation following the initiation or addition of opioids, along with at least two of the following criteria, including (1) 25% of bowel movements are hard, require significant effort, or are incomplete; anal obstruction exists and defecation needs manual assistance, and fewer than three spontaneous bowel movements appear per week; loose stools rarely occur without the use of laxatives. Rome criteria for OIC highlight the causal and temporal relationships between opioid use and constipation. Accordingly, the Italian 2021 Expert Consensus on OIC defines OIC as the presence of constipation symptoms that emerge or worsen over a period of at least 2 weeks [7].

## 3 Pathogenesis of OIC

### 3.1 Opioid Receptors

Opioids act through binding their opioid ligands with opioid receptors (MOR, DOR, KOR, NOPR, MRGPRX 1, MRGPRX 2, respectively) via signalling pathways [8]. In the classical GPCR signalling pathway, after the activation of GPCR and ligands, the binding G‐protein loses GDP and binds to GTP in the inactive state, then dissociates into the monomer of Gα and the dimer of Gβgγ and activates the downstream benefit molecules, respectively [9]. There are 16 types of Gα subtypes in mammals, and opioid receptors bind to the Gi/o family. After opioid receptor activation, Gai/o is produced to reduce cAMP level and inhibit voltage‐gated calcium channels (VGCC) [10]. Meanwhile, the GβGγ dimer is produced to regulate membrane and protein functions, including: (1) closing Ca^2+^ channels, (2) opening potassium ion channels (GIRK), (3) inhibiting protein complexes called vesicular SNAP receptors (SNARE) and reducing the release of pre‐protruding neurotransmitters [11]. G‐Protein‐coupled Receptor Kinase (GRK) promotes β‐arrestin recruitment and opioid receptor desensitisation by phosphorylating the C‐terminal tail of opioid receptors and intracellular rings (ICLs). This process avoids overreaction of the signalling pathway [8].

All subtypes of opioid receptors are present in the intestine, with MOR having the highest abundance. MOR is rich in immune cells located in the enteral muscle, the submucosal plexus and the longitudinal muscle of the ileum. DOR is distributed in both the large and small intestine, and the concentration of neuropeptide coexisting with DOR varies at different locations [12]. KOR is predominant in submucosal areas, Cajal stromal cells (interstitial cell of Cajal, ICCs) and myointerneurons.

### 3.2 OIC Mainly Arises as Opioids Impair Intestinal Motility

Opioid receptors in the enteric nervous system, ICCs and intestinal smooth muscle cells play a crucial role in regulating intestinal motility. The enteric nervous system, often referred to as the “brain” of the gut, controls the intestinal movements [13]. The ICCs function as both a “metronome” and “pacemaker” for intestinal smooth muscle cells or a “bridge” connecting intestinal neurons with intestinal smooth muscle cells [14]. The widespread distribution of opioid receptors in these components intensifies the impact of opioids on intestinal motility.

#### 3.2.1 Opioids Suppress Neuronal Signalling in the Intestines to Induce Intestinal Dysmotility

Signalling between neurons and effector cells is mainly undertaken by neurotransmitters [15]. In the classical signalling pathway, presynaptic neurons release neurotransmitters, which then bind to receptors on postsynaptic neurons. This binding activates ion channels, such as G‐protein‐coupled receptors (GPCR) and ligand‐gated channels, triggering the generation of an “action potential” [16]. The action potential propagates along the neuronal axon, ultimately reaching the distal end where voltage‐sensitive Ca^2+^ channels are activated. The influx of Ca^2+^ into the cell enables vesicles containing neurotransmitters to merge with the presynaptic cell membrane, leading to the release of neurotransmitters and the completion of information conduction. Various neurotransmitters, including acetylcholine, glutamate, γ‐aminobutyric acid (GABA), most monoamine neurotransmitters and neuropeptides, primarily act through GPCR receptors [17].

Inhibitory G‐proteins found on intestinal neurons, mainly μ‐opioid receptors (MOR) and δ‐opioid receptors (DOR), are associated with G‐proteins (GI proteins). After binding to opioid ligands, these receptors trigger the production of α‐GTP and γ‐GTP, along with GβGγ subunits that play significant roles in intracellular signalling pathways. Specifically, α‐GTP and γ‐GTP work to inhibit adenylate cyclase (AC), consequently reducing cyclic adenosine monophosphate (cAMP) levels and subsequently decreasing protein kinase A (PKA) activity [18, 19]. The decline in PKA activity leads to slow hyperpolarisation of enteric neurons (ENs), known as slow after‐hyperpolarisation (SAHP), which diminishes neuronal excitability and blocks neuronal signalling [19]. Additionally, GβGγ subunits modulate neuronal signalling by inhibiting calcium channels, opening potassium channels and suppressing SNARE proteins [11].

Voltage‐gated calcium channels (VGCCs), members of the transmembrane ion channel protein family, are activated upon the depolarisation of plasma membrane potential, leading to the influx of Ca^2+^ into the cell. The activity of VGCCs is inhibited under the activation of opioid receptors (including MOR, DOR, KOR and NOP) [20], leading to a decrease in intracellular Ca^2+^ concentration and neurotransmitter release.

In the intermuscular nerve plexus, excitatory motor neurons release neurotransmitters acetylcholine (Ash) and tachykinin (substance P), while inhibitory motor neurons release nitric oxide (NO), vasoactive intestinal polypeptide (VIP) and β‐nicotinamide adenine dinucleotide (β‐NAD) [21]. G‐protein‐coupled receptor (GPCR) has been identified as the receptor for Ash, with the VIP receptor also falling within this receptor class [22]. When exposed to opioids, GPCR binds to opioid ligands to inhibit neurotransmitter conduction, thereby interrupting signalling between neurons. Several studies have demonstrated that the binding of opioid ligands to receptors hinders the release and conduction of neurotransmitters, thus disrupting faecal function and intestinal transmission. Opioid ligands interfere in neurotransmitter activity to reduce gastrointestinal motility, as evidenced by a body of research [23].

#### 3.2.2 Opioids Decrease the Number of Kit+ICCs

ICC is closely associated with intestinal motility [24]. Reduced ICC abundance has been demonstrated in patients with gastrointestinal motility disorders and opioid‐exposed rabbit models, contributing to constipation development [24, 25]. While the mechanisms underlying ICC depletion in opioid‐induced constipation (OIC) remain unclear, it is established that ICCs originate from Kit‐positive mesenchymal precursor cells. Their differentiation requires Kit‐mediated cellular signalling, which experimental studies show can be inhibited by *LPS/TLR4/TNF‐α* pathways. Notably, TNF‐α‐induced ICC reduction has been shown to be reversible upon TNF‐α withdrawal [26]. These findings suggest that opioid‐induced intestinal inflammation may mediate ICC depletion in OIC patients. Omics analysis of faeces from humans and mice exposed to morphine revealed significant alterations in microbial composition, accompanied by elevated LPS production in murine faecal samples [27]. In vitro studies demonstrate that LPS/interferon‐induced inflammatory responses in intestinal cell clusters promote ICC phenotypic alterations and functional impairment without inducing apoptosis, potentially mediated through M1 macrophage polarization [28]. Mechanistically, exosomes secreted by M1‐polarised macrophages may regulate ICC quantity and function by targeting stem cell factor (SCF) signalling [29]. However, studies in Ednrb^−/−^ mice demonstrate that ICCs lacking Kit‐positive phenotypes can recover both morphology and pacemaker function through macrophage depletion or TNF‐α neutralization [30]. Crucially, extracellular vesicles (EVs)—key mediators of enterocyte‐immune cell communication—lose their immunomodulatory capacity following morphine treatment [31]. Emerging evidence suggests microbiota modulation can restore ICC populations [32], potentially via increased short‐chain fatty acid (SCFA) production and 5‐hydroxytryptamine (5‐HT) secretion [33]. While opioid‐induced ICC depletion appears linked to dysbiosis and intestinal inflammation, the precise mechanistic interplay requires further investigation.

#### 3.2.3 Opioids Cause Uncontrolled Contractions of Intestinal Smooth Muscle

Intestinal smooth muscle is regulated by excitatory motor neurons, inhibitory motor neurons and ICCs. Defecation is affected by many factors, such as intestinal muscle contraction, intestinal tension movement and intestinal lumen compliance [34, 35]. The decrease in neuronal excitability and ICC abundance fails the intestinal smooth muscle regulation system, which leads to the occurrence of OIC. We observed that opioid receptors are distributed on intestinal smooth muscle [12]. In mice exposed to morphine, contractility decreases in the proximal colon, ileum, jejunum and especially rectum [36]. Furthermore, in vitro experiments indicated that morphine and sufentanil increase the contractile tension of intestinal smooth muscle of rats [37]. Either directly or indirectly, opioids dysregulate intestinal smooth muscle movement, subsequently impeding the propulsion of the intestinal tract.

### 3.3 Opioids Cause Intestinal Microbiota Dysbiosis and Barrier Dysfunction to Induce OIC

Several animal studies have demonstrated that opioids can lead to intestinal dysbiosis, intestinal mucosal barrier dysfunction and the release of inflammatory cytokines in mice [38, 39, 40]. These changes disrupt the colonisation of intestinal microorganisms; meanwhile, intestinal flora metabolites may impair the functions of the enteric nervous system (ENS) and intestinal smooth muscle [41]. Additionally, opioids interact with inflammatory metabolites to worsen intestinal barrier dysfunction, thus establishing a vicious cycle ending up with OIC [35].

#### 3.3.1 Opioids Change Gut Microbiota Composition to Directly Reduce Intestinal Motility

Several studies have highlighted the significant changes in gut microbiota composition associated with opioid use. Clinical studies have shown that at the genus level, the abundances of Lactobacillus and anaerobic bacteria are notably altered in individuals using opioids when compared to a control group [42, 43]. Moreover, research has underscored the crucial role of enterobacteria metabolites in regulating intestinal motility. For instance, the metabolites produced by *Escherichia coli* can induce the secretion of serotonin (5‐hydroxytryptamine, 5‐HT) by activating enteroendocrine cells (EECs), thereby signalling the ENS and vagal nerve to enhance intestinal motility [44]. The metabolite short‐chain fatty acids (SCFA) derived from enterobacteria have been found to promote intestinal motility by stimulating 5‐HT3 receptors on vagal afferent fibers [45]. Interestingly, the activity of 5‐HT3 receptors is inhibited following the activation of mu‐opioid receptors (MOR) and kappa‐opioid receptors (KOR) [46]. It is plausible to infer that opioids may decrease intestinal SCFA concentrations to repress 5‐HT3 receptor activity, ultimately contributing to intestinal dysbiosis.

#### 3.3.2 Opioids Impair Intestinal Barrier to Reduce Intestinal Motility

The intestinal barrier, from the outside to the inside, is composed of mucus, gut microorganisms, defence proteins, intestinal epithelial cells and immune cells [47]. Any impairment of this barrier may cause bowel dysfunction [48]. Clinical studies have found a compensatory increase in goblet cells (GCs) and mucus in patients with functional constipation (FC), but their intestinal barrier function is not impaired [49]. In mice exposed to morphine, the tight junctions between intestinal epithelial cells exhibit a reduction, along with a decrease in goblet cell population and attenuation of the immune response, showing that opioid use is an independent risk factor for intestinal barrier function [50].

Loss of intestinal epithelial cells has been observed in patients undergoing long‐term methadone treatment. Clinical studies have demonstrated a decline in the integrity of the intestinal epithelium in these individuals, accompanied by a reduction in the abundance of *Akkermansia muciniphila* [51]. Additionally, *Akkermansia muciniphila* exerts beneficial effects through enhancing intestinal immunity, neural response and metabolism [52]. The maintenance of intestinal epithelial cells is contingent on the differentiation of crypt stem cells. Research has revealed that Notch signalling is pivotal in the differentiation and proliferation of crypt stem cells. However, exposure to morphine leads to a significant inhibition of the Notch pathway, further reducing the abundance of crypt stem cells [53].

The intestinal barrier acts as either a “buffer band” or a “protective layer” by blocking the passage of intestinal flora, pathogens and IECs, thereby promoting intestinal smoothness. This barrier is primarily composed of mucin and other immune mediators secreted by GCs [54]. GCs secrete their contents through two pathways: baseline secretion and stimulated secretion [55]. Baseline secretion is driven by intracellular spontaneous Ca^2+^ oscillation, which demands a full supply of ATP [56]. Intestinal flora serves as a source of ATP, as exemplified by the generation of butyrate through the fermentation of dietary fibre by the flora, followed by ATP production via β‐oxidation by IECs. Levels of Firmicutes and its metabolite butyrate decrease in mouse faeces following morphine intervention [57]. Additionally, mucus‐stimulated secretion by GCs is categorised into constitutive secretion and stimulated secretion, based on the source of stimulation. Under normal conditions, mucus secretion is primarily induced by intestinal peristalsis and the flow of intestinal contents [58]. Opioid‐induced inhibition of intestinal movement delays the transit of intestinal contents and decreases mucus secretion. Studies have indicated that opioids can lead to gut dysbiosis and intestinal inflammation [59]. Under inflammation, the majority of mucus particles in GCs are fused and released, a process known as complex exocytosis [60]. Sentinel goblet cells (senGCs), located at the opening of the colon, express Toll‐like receptors (TLRs) to identify pathogen‐associated molecular patterns (PAMPs). Exposure to opioids generates PAMPs that activate TLR 2/4/5, initiating the Nox/Duox pathway and enhancing the synthesis of reactive oxygen species (ROS). Activation of the NLRP6 inflammasome triggers the release of calcium ions from the endoplasmic reticulum (ER), inducing mucin exocytosis in senGCs. This process also activates mucin exocytosis in adjacent GCs, with senGC shedding occurring after mucus secretion. Short‐term opioid use minimally impacts senGCs due to compensatory GC secretion [61]. However, long‐term opioid use results in significant reduction of mucus secretion due to senGC shedding. Notably, water secretion in the intestinal mucus layer, dependent on Cl− channel activation, is reduced [62]. The release of acetylcholine (Ach) and vasoactive intestinal peptide (VIP) is inhibited when opioid ligands bind to mu‐opioid receptors (MORs) and delta opioid receptors (DORs) on secretory neurons in the intestinal submucosa. This inhibition reduces Cl− secretion, blocks Cl− channel activation and decreases intestinal moisture. The effects of short‐term opioid use on the intestinal mucus layer can be compensated by GC secretion, but long‐term use disrupts the composition of the mucus layer and water content, thus depriving the role of the mucus layer as a “buffer zone” and “protective layer.”

The role of intestinal immune cells in OIC has not been confirmed. Studies suggest that intestinal immune cells become activated following constipation [41]; the interaction between the intestinal immune system and the intestinal nervous system is the key to the maintenance of intestinal homeostasis [63], and the activation of intestinal immune cells may be caused by changes in intestinal flora [64]. Research indicates that MOR agonists suppress the immune response of macrophages and monocytes to LPS and reduce the cytotoxicity of NK cells, thereby promoting intestinal inflammation. This mechanism is closely related to β‐arrestins inhibiting the TLR signalling pathway [65, 66]. The composition of intestinal flora changes in patients treated with short‐ or long‐term morphine, mainly characterised by decreases in Bacteroidetes and Firmicutes and an increase in Proteobacteria [40, 67]. Regulatory T cells (Tregs) can inhibit intestinal inflammation through increasing the expression of opioid receptors in intestinal cells [68], as well as correcting intestinal motor dysregulation [69, 70]. The development and function of Treg depend on the production of gut microbial metabolite SCFA [71]. The production of SCFA, a metabolite of Bacteroidetes and Firmicutes, is reduced in patients after morphine treatment [72, 73]. Under the condition of constipation, a lower abundance of Lactobacillus in animals promotes the infiltration of macrophages and increases the release of inflammatory cytokines, which subsequently inhibit Treg differentiation and enhance colonic inflammation [74], thus forming a vicious cycle to further aggravate the intestinal motor dysbiosis. Bone morphogenetic protein 2 (BMP2), secreted by myographic macrophages, acts on the BMP receptor of intestinal neurons to induce the secretion of colony‐stimulating factor 1 (CSF1). CSF1, in turn, further stimulates J macrophages to secrete BMP2, a process that regulates intestinal smooth muscle contraction but is regulated by intestinal microbes [75]. However, exposure to opioids can induce the apoptosis of macrophages, reduce phagocytosis of macrophages and inhibit the recruitment of macrophages [76], thus impairing the immune function of macrophages so as to cause intestinal inflammation.

### 3.4 Opioids Indirectly Regulate Enteric Glial Cells to Promote OIC

**FIGURE 1:**
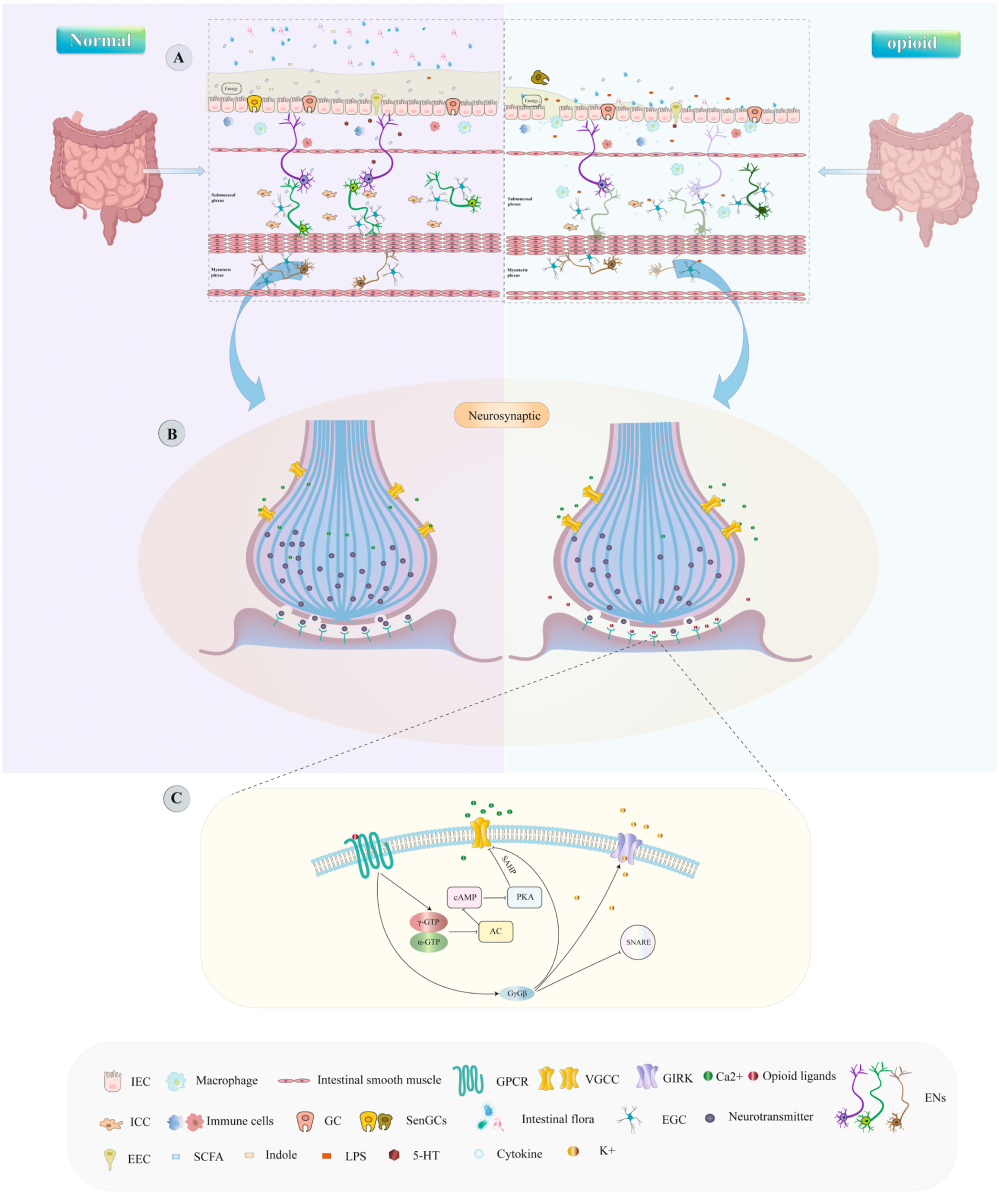
(A) Opioids cause abnormal physiological processes in the body. The secretion of mucins by the GCs in the intestine decreases, ultimately resulting in a thinner protective mucous layer. Concurrently, the composition of intestinal microbiota changes, leading to a decrease in the production of SCFAs and indole derivatives. These effects disrupt the energy supply to IECs and diminish the secretion of 5‐HT from EECs. Furthermore, the inflammatory response triggered by the influx of immune cells into the myenteric plexus causes macrophage infiltration, further exacerbating the impairment of intestinal barrier function. Additionally, the shift in intestinal flora composition promotes the production of LPS, which can induce neuronal cell damage in the intestine. This damage reduces the population of ICCs, leading to dysfunctional intestinal smooth muscle and subsequent dysmotility. Overall, exposure to opioids triggers a cascade of events that culminate in the impairment of intestinal barrier function and gastrointestinal dysmotility. (B) Inadequate Ca^2+^ influx of VGCC closure due to opioid receptor activation, highlighting blocked secretory neural mediators in anterior vesicles. The binding of opioid ligands to opioid receptors prevents the binding of neural medium and postsynaptic receptors, which affects nerve conduction. (C) α‐GTP, γ‐GTP and G β G γ generated as opioid receptors bind with opioid ligands are all involved in intracellular signalling, and α‐GTP and γ‐GTP affect VGCC opening by inhibiting the AC/cAMP/PKA pathway to generate SAHP. G β G γ affects neuronal signalling by blocking calcium channels, opening potassium channels and inhibiting SNARE.

Studies have shown that morphine causes OIC by increasing the expression of glial fibrillary acidic protein (GFAP) and the secretion of proinflammatory factors in enteric glial cells (EGCs). This effect can be reversed by silencing the MOR gene using siRNA, indicating the involvement of MOR in the development of OIC [77]. However, the specific mechanism of MOR in OIC remains unclear. Purinergic P2X receptor activity is significantly heightened in intestinal EGCs from morphine‐exposed mice and lipopolysaccharide (LPS) further amplifies adenosine‐5'‐triphosphate (ATP) signalling and enhances the expression of P2X4/7 receptors. Interestingly, in vitro studies have not identified any alterations in purinergic P2X receptor activity in EGCs [73]. ATP is known to induce enteric neuron death and disrupt intestinal motility through the EGCs‐purinergic‐connexin 43 (CX43) pathway [78]. Thus, opioid ligands may activate MOR in EGCs to trigger intestinal inflammation, while LPS disrupts gut microbiota to increase ATP release, implicating a role for ATP in driving OIC pathogenesis [79]. The colonic migrating motor complex (CMMC) propels faecal material to move through the colon, primarily driven by excitatory cholinergic neurons with EGC involvement. The NO/cGMP signalling pathway is essential for CMMC function [80]; NO is generated from L‐arginine by nitric oxide synthase (NOS), predominantly located in nitrergic neurons within the enteric muscle nerve plexus [81]. Additionally, MOR activation on EGCs by opioid ligands can induce intestinal inflammation, potentially linked to significant myenteric neuronal damage observed in a rat colitis model [82]. This damage may result from immune cell infiltration and increased activity of cytochrome P450 (CYP450)‐dependent inducible NOS (iNOS) and can be mitigated by iNOS inhibition (Figure 1) [83, 84].

## 4 Discussion

OIC as a common complication in cancer patients receiving opioid analgesia significantly impacts patients' economic status and quality of life [85]. Although clinical management options extend beyond first‐line traditional laxatives (e.g., polyethylene glycol, combination softener laxatives/stimulant laxatives) to include peripherally acting μ‐opioid receptor antagonists (PAMORAs), linaclotide, lubiprostone and prucalopride, therapeutic outcomes for OIC patients remain suboptimal [86]. By reviewing previous research findings, we identified that opioids may impair enteric motor neurons and ICCs to reduce gastrointestinal motility. Subsequent impairment of the gut microbiota and intestinal barrier function exacerbates inflammation and gastrointestinal motility disorders, ultimately resulting in OIC. The gut microbiota and their metabolites co‐work in the pathogenesis of OIC. However, current research is still not sufficient to uncover the complex underlying mechanisms. The reduction in the number of ICCs, which act as the “pacemakers” of gastrointestinal motility, may serve as a mechanism of OIC. The interaction between the gut bacteria and the enteric nervous systems is crucial for maintaining intestinal homeostasis, with SCFAs being vital for intestinal immune cells. Therefore, changes in the gut microbiota may contribute to dysregulation in the intestinal immune system.

In summary, the gut microbiota and their metabolites play a pivotal role in the pathogenesis of OIC and hold significant potential as therapeutic targets.

## Author Contributions

**Mengxue Liu:** conceptualization (equal), writing – original draft (equal). **Yedong Sheng:** conceptualization (equal), writing – original draft (equal). **Chunhui Jin:** resources (lead), supervision (lead), writing – review and editing (lead). **Lijuan Shen:** resources (supporting), writing – review and editing (supporting). **Shixiang Wu:** writing – original draft (supporting). **Yingrong He:** writing – original draft (supporting).

## Conflicts of Interest

The authors declare no conflicts of interest.

## Data Availability

Data sharing not applicable to this article as no datasets were generated or analysed during the current study.

## References

[1] Y. Wang , Q. Yan , C. Fan , et al., “Overview and Countermeasures of Cancer Burden in China,” Science China Life Sciences 66, no. 11 (2023): 2515–2526, 10.1007/s11427-022-2240-6. 37071289 PMC10111086

[2] R. Mesía , J. A. Virizuela Echaburu , J. Gómez , T. Sauri , G. Serrano , and E. Pujol , “Opioid‐Induced Constipation in Oncological Patients: New Strategies of Management,” Current Treatment Options in Oncology 20, no. 12 (2019): 91, 10.1007/s11864-019-0686-6. 31853656 PMC6920224

[3] J. Porta‐Sales , M. Nabal‐Vicuna , A. Vallano , et al., “Have We Improved Pain Control in Cancer Patients? A Multicenter Study of Ambulatory and Hospitalized Cancer Patients,” Journal of Palliative Medicine 18, no. 11 (2015): 923–932, 10.1089/jpm.2015.29002.jps. 26218494

[4] M. Rossi , G. Casale , D. Badiali , et al., “Opioid‐Induced Bowel Dysfunction: Suggestions From a Multidisciplinary Expert Board,” Supportive Care in Cancer 27, no. 11 (2019): 4083–4090, 10.1007/s00520-019-04688-2. 30778756 PMC6803581

[5] B. Hanson , S. M. Siddique , Y. Scarlett , and S. Sultan , “American Gastroenterological Association Institute Technical Review on the Medical Management of Opioid‐Induced Constipation,” Gastroenterology 156, no. 1 (2019): 229–253, 10.1053/j.gastro.2018.08.018. 30337104 PMC6685294

[6] W. Leppert , R. Zajaczkowska , and J. Wordliczek , “The Role of Oxycodone/Naloxone in the Management of Patients With Pain and Opioid‐Induced Constipation,” Expert Opinion on Pharmacotherapy 20, no. 5 (2019): 511–522, 10.1080/14656566.2018.1561863. 30625013

[7] R. De Giorgio , F. M. Zucco , G. Chiarioni , et al., “Management of Opioid‐Induced Constipation and Bowel Dysfunction: Expert Opinion of an Italian Multidisciplinary Panel,” Advances in Therapy 38, no. 7 (2021): 3589–3621, 10.1007/s12325-021-01766-y. 34086265 PMC8279968

[8] T. Che and B. L. Roth , “Molecular Basis of Opioid Receptor Signaling,” Cell 186, no. 24 (2023): 5203–5219, 10.1016/j.cell.2023.10.029. 37995655 PMC10710086

[9] V. V. Gurevich and E. V. Gurevich , “Gpcr Signaling Regulation: The Role of Grks and Arrestins,” Frontiers in Pharmacology 10 (2019): 125, 10.3389/fphar.2019.00125. 30837883 PMC6389790

[10] V. Dhyani , S. Gare , R. K. Gupta , S. Swain , K. V. Venkatesh , and L. Giri , “GPCR Mediated Control of Calcium Dynamics: A Systems Perspective,” Cellular Signalling 74 (2020): 109717, 10.1016/j.cellsig.2020.109717. 32711109 PMC7375278

[11] B. L. Roth , “Molecular Pharmacology of Metabotropic Receptors Targeted by Neuropsychiatric Drugs,” Nature Structural & Molecular Biology 26, no. 7 (2019): 535–544, 10.1038/s41594-019-0252-8. PMC6613815 31270468

[12] P. Mosińska , M. Zielińska , and J. Fichna , “Expression and Physiology of Opioid Receptors in the Gastrointestinal Tract,” Current Opinion in Endocrinology, Diabetes, and Obesity 23, no. 1 (2016): 3–10, 10.1097/MED.0000000000000219. 26702845

[13] M. Schemann , “Control of Gastrointestinal Motility by the “Gut Brain”—The Enteric Nervous System,” Journal of Pediatric Gastroenterology and Nutrition 41, no. S1 (2005): S4–S6, 10.1097/01.scs.0000180285.51365.55. 16131964

[14] J. D. Huizinga , A. Hussain , and J. Chen , “Interstitial Cells of Cajal and Human Colon Motility in Health and Disease,” American Journal of Physiology‐Gastrointestinal and Liver Physiology 321, no. 5 (2021): G552–G575, 10.1152/ajpgi.00264.2021. 34612070

[15] Y. Chen , L. Xiao , and J. Qiu , “Neuronomodulation of Excitable Neurons,” Neuroscience Bulletin 40, no. 1 (2024): 103–112, 10.1007/s12264-023-01095-w. 37584858 PMC10774251

[16] S. E. Hyman , “Neurotransmitters,” Current Biology 15, no. 5 (2005): R154–R158, 10.1016/j.cub.2005.02.037. 15753022

[17] S. Mochida , “Neurotransmitter Release Site Replenishment and Presynaptic Plasticity,” International Journal of Molecular Sciences 22, no. 1 (2020): 327, 10.3390/ijms22010327. 33396919 PMC7794938

[18] S. Chakrabarti , A. Chang , N. J. Liu , and A. R. Gintzler , “Chronic Opioid Treatment Augments Caveolin‐1 Scaffolding: Relevance to Stimulatory Mu‐Opioid Receptor Adenylyl Cyclase Signaling,” Journal of Neurochemistry 139, no. 5 (2016): 737–747, 10.1111/jnc.13852. 27726130 PMC5123915

[19] Z. Zhang and Z. Z. Pan , “Synaptic Mechanism for Functional Synergism Between Delta‐ and Mu‐Opioid Receptors,” Journal of Neuroscience 30, no. 13 (2010): 4735–4745, 10.1523/JNEUROSCI.5968-09.2010. 20357124 PMC2858689

[20] N. Weiss and G. W. Zamponi , “Opioid Receptor Regulation of Neuronal Voltage‐Gated Calcium Channels,” Cellular and Molecular Neurobiology 41, no. 5 (2021): 839–847, 10.1007/s10571-020-00894-3. 32514826 PMC11448596

[21] J. B. Furness , “Types of Neurons in the Enteric Nervous System,” Journal of the Autonomic Nervous System 81, no. 1–3 (2000): 87–96, 10.1016/s0165-1838(00)00127-2. 10869706

[22] Y. Xu , W. Feng , Q. Zhou , et al., “A Distinctive Ligand Recognition Mechanism by the Human Vasoactive Intestinal Polypeptide Receptor 2,” Nature Communications 13, no. 1 (2022): 2272, 10.1038/s41467-022-30041-z. PMC9046186 35477937

[23] J. J. Galligan and C. Sternini , “Insights Into the Role of Opioid Receptors in the Gi Tract: Experimental Evidence and Therapeutic Relevance,” Handbook of Experimental Pharmacology 239 (2017): 363–378, 10.1007/164_2016_116. 28204957 PMC6310692

[24] D. Foong , J. Zhou , A. Zarrouk , V. Ho , and M. D. O'Connor , “Understanding the Biology of Human Interstitial Cells of Cajal in Gastrointestinal Motility,” International Journal of Molecular Sciences 21, no. 12 (2020): 4540, 10.3390/ijms21124540. 32630607 PMC7352366

[25] H. Yang , H. Luo , and Y. H. Li , “Effects of Epidural Infusion of Morphine Combined With Small‐Dose Naloxone on Gastrointestinal Interstitial Cells of Cajal in Rabbits,” European Review for Medical and Pharmacological Sciences 23, no. 6 (2019): 2596–2601, 10.26355/eurrev_201903_17409. 30964188

[26] S. Torihashi , K. Nishi , Y. Tokutomi , T. Nishi , S. Ward , and K. M. Sanders , “Blockade of Kit Signaling Induces Transdifferentiation of Interstitial Cells of Cajal to a Smooth Muscle Phenotype,” Gastroenterology 117, no. 1 (1999): 140–148, 10.1016/S0016-5085(99)70560-3. 10381920

[27] U. Kolli , R. Jalodia , S. Moidunny , et al., “Multi‐Omics Analysis Revealing the Interplay Between Gut Microbiome and the Host Following Opioid Use,” Gut Microbes 15, no. 2 (2023): 2246184, 10.1080/19490976.2023.2246184. 37610102 PMC10448978

[28] N. Kaji , K. Horiguchi , S. Iino , et al., “Nitric Oxide‐Induced Oxidative Stress Impairs Pacemaker Function of Murine Interstitial Cells of Cajal During Inflammation,” Pharmacological Research 111 (2016): 838–848, 10.1016/j.phrs.2016.07.030. 27468647

[29] S. U. Xu , J. Zhai , K. E. Xu , et al., “M1 Macrophages‐Derived Exosomes miR‐34c‐5p Regulates Interstitial Cells of Cajal Through Targeting SCF,” Journal of Biosciences 46 (2021): 90. 34544909

[30] X. Chen , X. Meng , H. Zhang , et al., “Intestinal Proinflammatory Macrophages Induce a Phenotypic Switch in Interstitial Cells of Cajal,” Journal of Clinical Investigation 130, no. 12 (2020): 6443–6456, 10.1172/JCI126584. 32809970 PMC7685750

[31] Y. Zhang , Y. Yan , J. Meng , M. Girotra , S. Ramakrishnan , and S. Roy , “Immune Modulation Mediated by Extracellular Vesicles of Intestinal Organoids Is Disrupted by Opioids,” Mucosal Immunology 14, no. 4 (2021): 887–898, 10.1038/s41385-021-00392-9. 33854193 PMC8225561

[32] Y. Guo , L. Song , Y. Huang , et al., “Latilactobacillus Sakei Furu2019 and Stachyose as Probiotics, Prebiotics, and Synbiotics Alleviate Constipation in Mice,” Frontiers in Nutrition 9 (2022): 1039403, 10.3389/fnut.2022.1039403. 36687730 PMC9849682

[33] S. Cheng , H. Li , Y. Ding , et al., “The Probiotic Combination of Lacticaseibacillus Paracasei JY062 and *lactobacillus gasseri* JM1 Alleviates Gastrointestinal Motility Disorder via Improving Gut Microbiota,” Nutrients 15, no. 4 (2023): 839, 10.3390/nu15040839. 36839197 PMC9958595

[34] Q. Liu , Y. Luo , and X. Ke , “Interaction Between the Gut Microbiota and Intestinal Motility,” Evidence‐Based Complementary and Alternative Medicine 2022 (2022): 1–5, 10.1155/2022/3240573. PMC9681544 36425261

[35] Z. Zheng , J. Tang , Y. Hu , and W. Zhang , “Role of Gut Microbiota‐Derived Signals in the Regulation of Gastrointestinal Motility,” Frontiers in Medicine 9 (2022): 961703, 10.3389/fmed.2022.961703. 35935766 PMC9354785

[36] H. Ono , A. Nakamura , K. Matsumoto , S. Horie , G. Sakaguchi , and T. Kanemasa , “Circular Muscle Contraction in the Mice Rectum Plays a Key Role in Morphine‐Induced Constipation,” Neurogastroenterology and Motility 26, no. 10 (2014): 1396–1407, 10.1111/nmo.12387. 25041353

[37] X. Bian , R. Zhou , Y. Yang , et al., “Divergent Effect of Dezocine, Morphine and Sufentanil on Intestinal Motor Function in Rats,” International Journal of Medical Sciences 12, no. 11 (2015): 848–852, 10.7150/ijms.12616. 26640403 PMC4643074

[38] S. Banerjee , G. Sindberg , F. Wang , et al., “Opioid‐Induced Gut Microbial Disruption and Bile Dysregulation Leads to Gut Barrier Compromise and Sustained Systemic Inflammation,” Mucosal Immunology 9, no. 6 (2016): 1418–1428, 10.1038/mi.2016.9. 26906406 PMC4996771

[39] L. Zhang , J. Meng , Y. Ban , et al., “Morphine Tolerance Is Attenuated in Germfree Mice and Reversed by Probiotics, Implicating the Role of Gut Microbiome,” Proceedings of the National Academy of Sciences of the United States of America 116, no. 27 (2019): 13523–13532, 10.1073/pnas.1901182116. 31209039 PMC6613141

[40] F. Wang , J. Meng , L. Zhang , T. Johnson , C. Chen , and S. Roy , “Morphine Induces Changes in the Gut Microbiome and Metabolome in a Morphine Dependence Model,” Scientific Reports 8, no. 1 (2018): 3515–3596, 10.1038/s41598-018-21915-8. 29483538 PMC5827657

[41] R. Pan , L. Wang , X. Xu , et al., “Crosstalk Between the Gut Microbiome and Colonic Motility in Chronic Constipation: Potential Mechanisms and Microbiota Modulation,” Nutrients 14, no. 18 (2022): 3704, 10.3390/nu14183704. 36145079 PMC9505360

[42] H. Wang , J. Luo , X. Chen , et al., “Clinical Observation of the Effects of Oral Opioid on Inflammatory Cytokines and Gut Microbiota in Patients With Moderate to Severe Cancer Pain: A Retrospective Cohort Study,” Pain and Therapy 11, no. 2 (2022): 667–681, 10.1007/s40122-022-00386-w. 35435623 PMC9098777

[43] E. Dimidi , S. S. Mark , and K. Whelan , “Probiotics and Constipation: Mechanisms of Action, Evidence for Effectiveness and Utilisation by Patients and Healthcare Professionals,” Proceedings of the Nutrition Society 79, no. 1 (2020): 147–157, 10.1017/S0029665119000934. 31262376

[44] L. Ye , M. Bae , C. D. Cassilly , et al., “Enteroendocrine Cells Sense Bacterial Tryptophan Catabolites to Activate Enteric and Vagal Neuronal Pathways,” Cell Host & Microbe 29, no. 2 (2021): 179–196, 10.1016/j.chom.2020.11.011. 33352109 PMC7997396

[45] S. Fried , E. Wemelle , P. D. Cani , and C. Knauf , “Interactions Between the Microbiota and Enteric Nervous System During Gut‐Brain Disorders,” Neuropharmacology 197 (2021): 108721, 10.1016/j.neuropharm.2021.108721. 34274348

[46] A. J. Fox and I. K. Morton , “Mu and Kappa Opioid Receptor Modulation of 5‐HT3 and NK‐3 Receptor‐Evoked Release of Acetylcholine From the Guinea‐Pig Ileum Myenteric Plexus,” Naunyn‐Schmiedeberg's Archives of Pharmacology 344, no. 1 (1991): 8–15, 10.1007/BF00167377. 1723152

[47] N. Di Tommaso , A. Gasbarrini , and F. R. Ponziani , “Intestinal Barrier in Human Health and Disease,” International Journal of Environmental Research and Public Health 18, no. 23 (2021): 12836, 10.3390/ijerph182312836. 34886561 PMC8657205

[48] V. D'Antongiovanni , C. Pellegrini , M. Fornai , et al., “Intestinal Epithelial Barrier and Neuromuscular Compartment in Health and Disease,” World Journal of Gastroenterology 26, no. 14 (2020): 1564–1597, 10.3748/wjg.v26.i14.1564. 32327906 PMC7167418

[49] J. Wang , W. Wei , D. Zhao , et al., “Intestinal Mucosal Barrier in Functional Constipation: Dose It Change?,” World Journal of Clinical Cases 10, no. 19 (2022): 6385–6398, 10.12998/wjcc.v10.i19.6385. 35979313 PMC9294902

[50] F. Wang , J. Meng , L. Zhang , and S. Roy , “Opioid Use Potentiates the Virulence of Hospital‐Acquired Infection, Increases Systemic Bacterial Dissemination and Exacerbates Gut Dysbiosis in a Murine Model of *citrobacter rodentium* Infection,” Gut Microbes 11, no. 2 (2020): 172–190, 10.1080/19490976.2019.1629237. 31379246 PMC7053978

[51] A. Cruz‐Lebron , R. Johnson , C. Mazahery , et al., “Chronic Opioid Use Modulates Human Enteric Microbiota and Intestinal Barrier Integrity,” Gut Microbes 13, no. 1 (2021): 1946368, 10.1080/19490976.2021.1946368. 34313547 PMC8317955

[52] Q. Zhao , J. Yu , Y. Hao , et al., “ *Akkermansia muciniphila* Plays Critical Roles in Host Health,” Critical Reviews in Microbiology 49, no. 1 (2023): 82–100, 10.1080/1040841X.2022.2037506. 35603929

[53] J. Meng , S. Banerjee , L. Zhang , et al., “Opioids Impair Intestinal Epithelial Repair in HIV‐Infected Humanized Mice,” Frontiers in Immunology 10 (2020): 2999, 10.3389/fimmu.2019.02999. 32010125 PMC6978907

[54] M. E. Johansson and G. C. Hansson , “Immunological Aspects of Intestinal Mucus and Mucins,” Nature Reviews Immunology 16, no. 10 (2016): 639–649, 10.1038/nri.2016.88. PMC6435297 27498766

[55] B. Dolan , A. Ermund , B. Martinez‐Abad , M. E. V. Johansson , and G. C. Hansson , “Clearance of Small Intestinal Crypts Involves Goblet Cell Mucus Secretion by Intracellular Granule Rupture and Enterocyte Ion Transport,” Science Signaling 15, no. 752 (2022): eabl5848, 10.1126/scisignal.abl5848. 36126118 PMC9749883

[56] R. Wollman and T. Meyer , “Coordinated Oscillations in Cortical Actin and Ca^2+^ Correlate With Cycles of Vesicle Secretion,” Nature Cell Biology 14, no. 12 (2012): 1261–1269, 10.1038/ncb2614. 23143397 PMC3777337

[57] K. H. Muchhala , P. S. Kallurkar , M. Kang , et al., “The Role of Morphine‐Induced Impairment of Intestinal Epithelial Antibacterial Activity in Dysbiosis and Its Impact on the Microbiota‐Gut‐Brain Axis,” FASEB J 38, no. 8 (2024): e23603, 10.1096/fj.202301590RR. 38648368 PMC11047137

[58] Y. Jiang , J. Song , Y. Xu , et al., “Piezo1 Regulates Intestinal Epithelial Function by Affecting the Tight Junction Protein Claudin‐1 via the Rock Pathway,” Life Sciences 275 (2021): 119254, 10.1016/j.lfs.2021.119254. 33636174

[59] R. E. Gicquelais , A. Bohnert , L. Thomas , and B. Foxman , “Opioid Agonist and Antagonist Use and the Gut Microbiota: Associations Among People in Addiction Treatment,” Scientific Reports 10, no. 1 (2020): 19471, 10.1038/s41598-020-76570-9. 33173098 PMC7655955

[60] G. M. Birchenough , M. E. Johansson , J. K. Gustafsson , J. H. Bergstrom , and G. C. Hansson , “New Developments in Goblet Cell Mucus Secretion and Function,” Mucosal Immunology 8, no. 4 (2015): 712–719, 10.1038/mi.2015.32. 25872481 PMC4631840

[61] G. M. Birchenough , E. E. Nystrom , M. E. Johansson , and G. C. Hansson , “A Sentinel Goblet Cell Guards the Colonic Crypt by Triggering Nlrp6‐Dependent Muc2 Secretion,” Science 352, no. 6293 (2016): 1535–1542, 10.1126/science.aaf7419. 27339979 PMC5148821

[62] G. Fei , K. Raehal , S. Liu , et al., “Lubiprostone Reverses the Inhibitory Action of Morphine on Intestinal Secretion in Guinea Pig and Mouse,” Journal of Pharmacology and Experimental Therapeutics 334, no. 1 (2010): 333–340, 10.1124/jpet.110.166116. 20406855 PMC2912047

[63] X. Bai , G. De Palma , E. Boschetti , et al., “Vasoactive Intestinal Polypeptide Plays a Key Role in the Microbial‐Neuroimmune Control of Intestinal Motility,” Cellular and Molecular Gastroenterology and Hepatology 17, no. 3 (2024): 383–398, 10.1016/j.jcmgh.2023.11.012. 38061549 PMC10825443

[64] L. Y. Wan , Z. J. Chen , N. P. Shah , and H. El‐Nezami , “Modulation of Intestinal Epithelial Defense Responses by Probiotic Bacteria,” Critical Reviews in Food Science and Nutrition 56, no. 16 (2016): 2628–2641, 10.1080/10408398.2014.905450. 25629818

[65] P. Zhang , M. Yang , C. Chen , L. Liu , X. Wei , and S. Zeng , “Toll‐Like Receptor 4 (TLR4)/opioid Receptor Pathway Crosstalk and Impact on Opioid Analgesia, Immune Function, and Gastrointestinal Motility,” Frontiers in Immunology 11 (2020): 1455, 10.3389/fimmu.2020.01455. 32733481 PMC7360813

[66] D. P. Maher , D. Walia , and N. M. Heller , “Morphine Decreases the Function of Primary Human Natural Killer Cells by Both TLR4 and Opioid Receptor Signaling,” Brain, Behavior, and Immunity 83 (2020): 298–302, 10.1016/j.bbi.2019.10.011. 31626971

[67] M. Kang , R. A. Mischel , S. Bhave , et al., “The Effect of Gut Microbiome on Tolerance to Morphine Mediated Antinociception in Mice,” Scientific Reports 7 (2017): 42658, 10.1038/srep42658. 28211545 PMC5314392

[68] O. Pol , F. Alameda , and M. M. Puig , “Inflammation Enhances Mu‐Opioid Receptor Transcription and Expression in Mice Intestine,” Molecular Pharmacology 60, no. 5 (2001): 894–899, 10.1124/mol.60.5.894. 11641416

[69] T. Der , P. Bercik , G. Donnelly , et al., “Interstitial Cells of Cajal and Inflammation‐Induced Motor Dysfunction in the Mouse Small Intestine,” Gastroenterology 119, no. 6 (2000): 1590–1599, 10.1053/gast.2000.20221. 11113080

[70] L. Carrascal , M. D. Vazquez‐Carretero , P. Garcia‐Miranda , et al., “Acute Colon Inflammation Triggers Primary Motor Cortex Glial Activation, Neuroinflammation, Neuronal Hyperexcitability, and Motor Coordination Deficits,” International Journal of Molecular Sciences 23, no. 10 (2022): 5347, 10.3390/ijms23105347. 35628158 PMC9141031

[71] C. Martin‐Gallausiaux , L. Marinelli , H. M. Blottière , P. Larraufie , and N. Lapaque , “SCFA: Mechanisms and Functional Importance in the Gut,” Proceedings of the Nutrition Society 80, no. 1 (2021): 37–49, 10.1017/S0029665120006916. 32238208

[72] S. Fujisaka , Y. Watanabe , and K. Tobe , “The Gut Microbiome: A Core Regulator of Metabolism,” Journal of Endocrinology 256, no. 3 (2023): e220111, 10.1530/JOE-22-0111. 36458804 PMC9874984

[73] K. Nomura , D. Ishikawa , K. Okahara , et al., “Bacteroidetes Species Are Correlated With Disease Activity in Ulcerative Colitis,” Journal of Clinical Medicine 10, no. 8 (2021): 1749, 10.3390/jcm10081749. 33920646 PMC8073534

[74] Y. Zhang , F. Song , M. Yang , et al., “Gastrointestinal Dysmotility Predisposes to Colitis Through Regulation of Gut Microbial Composition and Linoleic Acid Metabolism,” Advanced Science 11 (2024): e2306297, 10.1002/advs.202306297. 38477534 PMC11132037

[75] C. C. Bain and A. Schridde , “Origin, Differentiation, and Function of Intestinal Macrophages,” Frontiers in Immunology 9 (2018): 2733, 10.3389/fimmu.2018.02733. 30538701 PMC6277706

[76] S. Wen , Y. Jiang , S. Liang , Z. Cheng , X. Zhu , and Q. Guo , “Opioids Regulate the Immune System: Focusing on Macrophages and Their Organelles,” Frontiers in Pharmacology 12 (2022): 814241, 10.3389/fphar.2021.814241. 35095529 PMC8790028

[77] H. Gao , Y. Zhang , Y. Li , et al., “Mu‐Opioid Receptor‐Mediated Enteric Glial Activation Is Involved in Morphine‐Induced Constipation,” Molecular Neurobiology 58, no. 7 (2021): 3061–3070, 10.1007/s12035-021-02286-0. 33624141

[78] G. Burnstock , K. A. Jacobson , and F. L. Christofi , “Purinergic Drug Targets for Gastrointestinal Disorders,” Current Opinion in Pharmacology 37 (2017): 131–141, 10.1016/j.coph.2017.10.011. 29149731 PMC5859324

[79] L. Seguella and B. D. Gulbransen , “Enteric Glial Biology, Intercellular Signalling and Roles in Gastrointestinal Disease,” Nature Reviews Gastroenterology & Hepatology 18, no. 8 (2021): 571–587, 10.1038/s41575-021-00423-7. 33731961 PMC8324524

[80] K. Beck , B. Voussen , A. Reigl , et al., “Cell‐Specific Effects of Nitric Oxide on the Efficiency and Frequency of Long Distance Contractions in Murine Colon,” Neurogastroenterology and Motility 31, no. 6 (2019): e13589, 10.1111/nmo.13589. 30947401

[81] N. Bódi , Z. Szalai , and M. Bagyánszki , “Nitrergic Enteric Neurons in Health and Disease—Focus on Animal Models,” International Journal of Molecular Sciences 20, no. 8 (2019): 2003, 10.3390/ijms20082003. 31022832 PMC6515552

[82] P. Talapka , “Alleviated Mucosal and Neuronal Damage in a Rat Model of Crohn's Disease,” World Journal of Gastroenterology 20, no. 44 (2014): 16690–16697, 10.3748/wjg.v20.i44.16690. 25469038 PMC4248213

[83] S. Venkataramana , S. Lourenssen , K. G. Miller , and M. G. Blennerhassett , “Early Inflammatory Damage to Intestinal Neurons Occurs via Inducible Nitric Oxide Synthase,” Neurobiology of Disease 75 (2015): 40–52, 10.1016/j.nbd.2014.12.014. 25562655

[84] F. Turco , G. Sarnelli , C. Cirillo , et al., “Enteroglial‐Derived S100B Protein Integrates Bacteria‐Induced Toll‐Like Receptor Signalling in Human Enteric Glial Cells,” Gut 63, no. 1 (2014): 105–115, 10.1136/gutjnl-2012-302090. 23292665

[85] M. F. Butt , G. Isherwood , T. Lewis‐Lawson , et al., “Clinical Characteristics and Outcomes of Patients With Rome IV Functional Dyspepsia Who Consume Opioids: A Real‐World Study,” Neurogastroenterology and Motility (2025): e15019, 10.1111/nmo.15019. 40017096 PMC12163204

[86] A. Davies , N. Fagan , J. Gonzalez‐Barboteo , et al., “Inadequate Management of Opioid‐Induced Constipation in European Cancer Pain Patients: Results of a Real‐World, Multicentre, Observational Study (“e‐Stoic”),” Support Care Cancer 32, no. 10 (2024): 701, 10.1007/s00520-024-08898-1. 39367106

